# Oral administration of E-type prostanoid (EP) 1 receptor antagonist suppresses carcinogenesis and development of prostate cancer via upregulation of apoptosis in an animal model

**DOI:** 10.1038/s41598-021-99694-y

**Published:** 2021-10-13

**Authors:** Masahito Masato, Yasuyoshi Miyata, Hiroki Kurata, Hidenori Ito, Kensuke Mitsunari, Akihiro Asai, Yuichiro Nakamura, Kyohei Araki, Yuta Mukae, Tsuyoshi Matsuda, Junki Harada, Tomohiro Matsuo, Kojiro Ohba, Hideki Sakai

**Affiliations:** Department of Urology, Nagasaki University Graduate School of Biomedical Sciences, 1-7-1 Sakamoto, Nagasaki, 852-8501 Japan

**Keywords:** Cancer, Oncology, Urology

## Abstract

Prostaglandin E2 plays an important role in carcinogenesis and malignant potential of prostate cancer (PC) cells by binding to its specific receptors, E-type prostanoid (EP) receptors. However, anti-carcinogenic effects of the EP receptor antagonist are unclear. In this study, we used a mouse model of PC. The mice were provided standard feed (control) or feed containing the EP1 receptor antagonist and were sacrificed at 10, 15, 30, and 52 weeks of age. Apoptosis was evaluated by immunohistochemical analysis using a cleaved caspase-3 assay. The incidence of cancer in the experimental group was significantly lower than that in the control group at 15, 30, and 52 weeks of age. The percentage of poorly differentiated PC cells was significantly lower in the experimental group than in the control group at 30 and 52 weeks of age. The percentage of apoptotic cells in the experimental group was significantly higher than that in the control group at 15, 30, and 52 weeks of age. These findings indicate that feeding with the addition of EP1 receptor antagonist delayed PC progression via the upregulation of apoptosis. We suggest that the EP1 receptor antagonist may be a novel chemopreventive agent for PC.

## Introduction

Prostate cancer (PC) is the most common malignancy in men. Treatments, including surgery, hormonal therapy, chemotherapy, and radiotherapy, are performed for PC patients according to their clinicopathological features and background^1–3^. In addition, active surveillance of patients with favorable- and intermediate-risk PC has been reported as clinically benefical^3,4^. Conservative treatments for a PC patient, including active surveillance, can minimize the risk of adverse events, maintain quality of life, and prevent further medical intervention. Thus, information on suppression methods of malignant potential and tumor growth is critical, and it contributes to the treatment strategies aimed at both improving prognosis and maintaining quality of life in patients with PC.

Prostaglandin E2 (PGE2) is a strong mediator of various pathological conditions including cancers^5,6^. Cyclooxygenase (COX)-2 plays a crucial role in the metabolism of arachidonic acid to PGE2. Therefore, COX-2 is well known to be positively associated with carcinogenesis, malignant aggressiveness, and poor outcomes in many types of malignancies^7–9^. However, the pathological activity of PGE2 is not strictly dependent on COX-2; factors other than COX-2 regulate PGE2 production^10,11^. Briefly, although COX-2 inhibitors, including non-steroid anti-inflammatory drugs, are reported to act as tumor suppressors via regulation of PGE2 in many types of cancers^12,13^, COX-2 inhibitors do not always inhibit the pathological activities of PGE2. However, we must also consider that binding of PGE2 to its specific receptor, E-prostanoid receptor (EP), is essential to the pathophysiological functioning of PGE2^14^. The EP receptor family consists of four isoforms (EP1–4 receptor), and the interactions between PGE2 and EP receptors in malignancies vary depending on cell type and tumor microenvironment^7,15,16^.

Many investigators have suggested that the PGE2/EP receptor axes in PC play important pathological roles in malignant potential and tumor growth^16–20^. While increased expression of the EP1, EP2, and EP4 receptors and reduced expression of the EP3 receptor have been reported in PC tissues, the detailed pathological significance of each EP receptor in PC tissues is not fully understood^17–19^. In addition, there is little information on the efficacy and safety of chemopreventive and treatment strategies using anti-EP receptor agents in PC by in vivo studies. In a previous study, we showed that EP1, EP2, and EP4 receptors play crucial roles in carcinogenesis in patients with hormone sensitive PC^17^. In addition, EP1 receptor expression was shown to be positively associated with tumor grade and TNM stage^17^. Based on these results, we hypothesized that blocking of the EP1 receptor leads to suppression of carcinogenesis and of tumor growth in PC in vivo. The main aim of this study was to test this hypothesis using a PC mouse model that showed close-to-human kinetics of tumor development. In addition, the influence of the EP1 receptor antagonist on apoptosis in PC cells in the same mouse tissues was assessed.

## Materials and methods

### Animals

The knock-in mouse adenocarcinoma prostate (KIMAP) model was used in this study. There is no naturally occurring prostate tumor murine model, and we established a KIMAP model by knock-in technology; in brief, we used the viral SV40 Tag to target the prostate tissue-specific gene *PSP94,* which is translated to the 94-amino-acid prostate secretory protein^21^. The pathology and tumor progression kinetics of PC in the KIMAP model are similar to those of human PC, and this model has previously been used to evaluate the pathological roles of cancer-related factors and anti-cancer effects of various foods^22–24^. Indeed, hematoxylin–eosin (HE) staining, used to demonstrate similarities between the model and human pathologies, was used to clearly distinguish between prostatic intraepithelial neoplasia (PIN), well- and moderately differentiated PC, and poorly differentiated PC in the KIMAP model. The detailed information on rearing environment, anesthesia, and welfare is described in our previous reports^23,25^. In this study, a total of 120 mice were used (15 mice per timepoint for each of the experimental and control groups).

All animal experiments were performed according to the Guidelines for Animal Experiments of Nagasaki University, and the protocol was approved by the Regulations of Animal Care and Use Committee of Nagasaki University. We confirmed that this study is reported in accordance with ARRIVE guidelines.

### Food preparation

The selective EP1 receptor antagonist ONO-8713 (provided by ONO Pharmaceuticals, Osaka, Japan) was orally administered through feed. In the experimental group, ONO-8713 was mixed with standard feed (AIN-76A, CLEA Japan, Inc., Tokyo, Japan) to a final concentration of 1000 ppm. This concentration was selected based on a previous study using this EP1 receptor antagonist in a mouse model^26^. Feed with EP1 receptor antagonist was administered from 8 weeks of age, and we confirmed that the total quantities of feed consumed were similar for the control and experimental groups each week.

### Tissue collection and analyses

**Figure 1:**
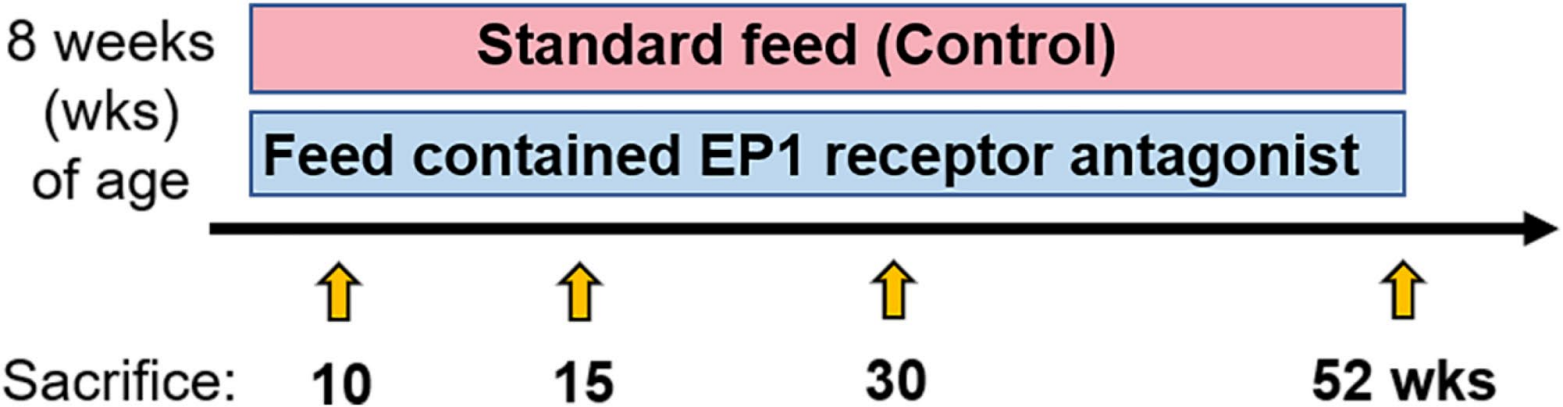
Summary of the animal experiments. Mice were fed with or without EP1 receptor antagonist from 8 weeks of age, and mice were sacrificed at 10, 15, 30, and 52 weeks of age.

Mice were sacrificed and prostate tissues were collected at 10, 15, 30, and 52 weeks of age. To diagnose and determine the extent of cancer cell differentiation, we stained the harvested prostate tissues with hematoxylin and eosin (H&E) and visualized. Specifically, we analyzed the ventral prostate lobe and dorsolateral prostate lobes of the KIMAPs. A schematic of the study protocol is shown in Fig. 1. A previous study showed that PIN, well- and moderately differentiated PC, and poorly differentiated PC were clearly diagnosed via H&E staining^22^. Other studies have detailed the specific diagnostic criteria of poorly differentiated PC^27,28^. In short, the architectural patterns of adenocarcinoma observed were scored according to five histological grades; a tissue was classified as poorly differentiated PC if cribriform masses with ragged, invading edges and fused glands were detected. In this study, adenocarcinoma was defined as a PC, whereas all grades of PIN, including high grade, were not.

EP1 receptor immunoreactivity in PC tissues was examined via an immunohistochemical technique using an anti-EP1 receptor rabbit-polyclonal antibody (Cayman Chemical, Ann Arbor, MI). The specificity of this anti-EP1 antibody and methods are described in a previous study^17^. The apoptotic index (AI) was calculated by anti-cleaved caspase-3 (Asp 175) (R&D Systems, Minneapolis, MN) according to our previous report^23,29^. Briefly, 5-µm-thick formalin-fixed and paraffin-embedded sections were deparaffinized and re-hydrated; antigen retrieval was performed using 0.01 M sodium citrate buffer (pH 6.0), and endogenous peroxidase activity was inhibited using 3% hydrogen peroxide. Following tissue incubation with primary antibody, specimens were treated with Dako EnVision + ™ Peroxidase (DakoCytomation, Carpinteria, CA). Lastly, we calculated the apoptotic index (AI) as a percentage of cleaved caspase-3-positive cancer cells / all cancer cells.

### Statistical analyses

All data were expressed as median and interquartile range (IQR). The Mann–Whitney U test was used to compare continuous variables. Kaplan–Meier survival curves and the log-rank test were performed for survival analysis. A significance was defined as *p* < 0.05. All statistical analyses were performed by statistical package StatView for Windows (Version 5.0, Abacus Concept, Berkeley, CA).

## Results

### Histology changes and characteristics

**Figure 2:**
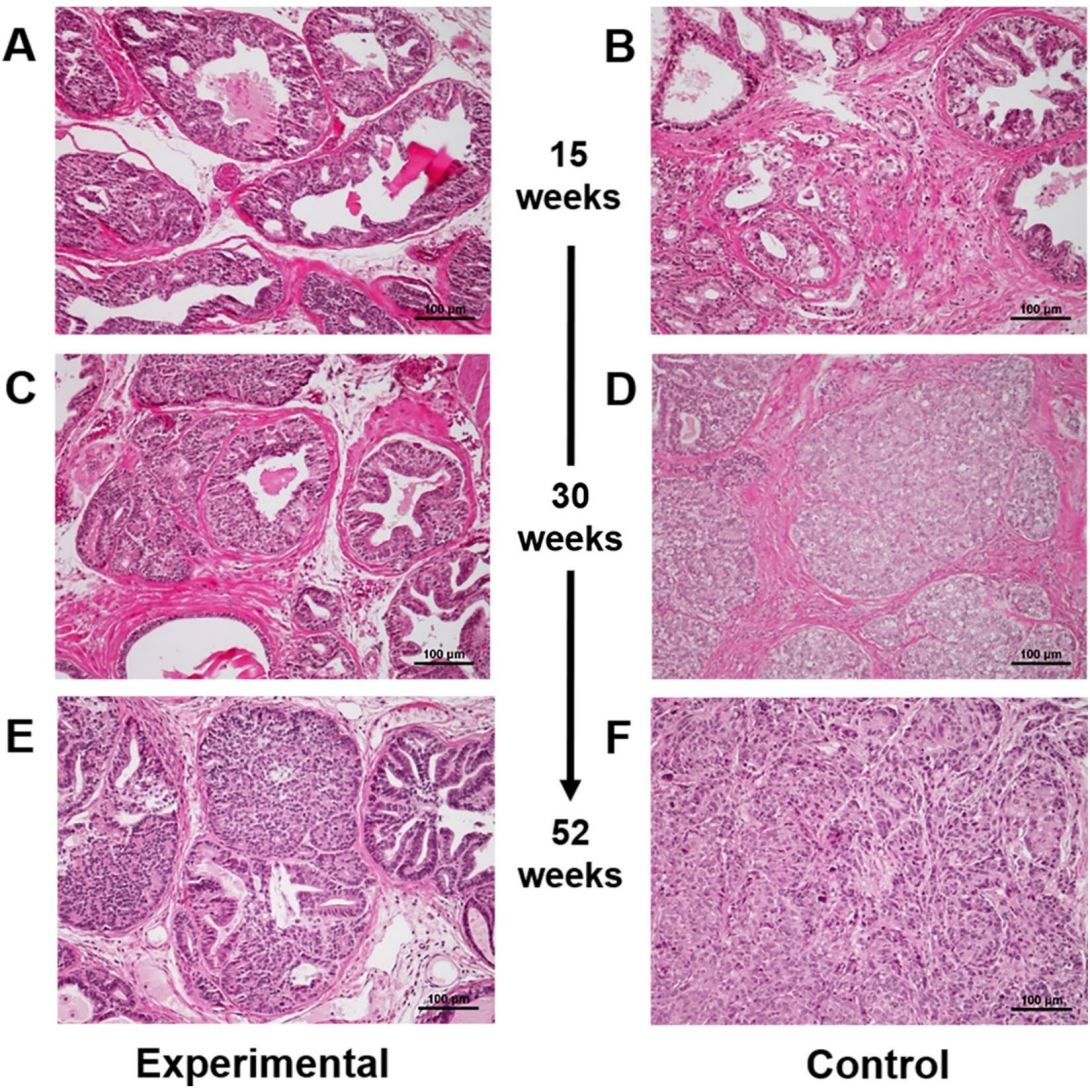
Hematoxylin and eosin-stained tissues at 15, 30, and 52 weeks of age in experimental (**A**, **C**, and **E**, respectively) and control mice (**B**, **D**, **F**, respectively). Magnification × 200.

Firstly, we confirmed that expression of the EP1 receptor was detected in all PC tissues of both the experimental and control groups. In addition, we noted that the receptor expression in cancer cells was higher than that in non-cancer glands (Figure S1). In addition, carcinogenic changes were found in ventral prostate lobe, but not in dorsolateral prostate lobes. PC cells were not detected in either group at 10 weeks of age. At 15 weeks of age, cancer cells were relatively rare in the experimental group (Fig. 2A); however, carcinogenic changes in the prostate glands were found in the control group (Fig. 2B). Indeed, the median/IQR of the percentage of cancer cells in the experimental group (11.0/9.7–12.2%) was significantly lower (p < 0.001) than in the control group (50.7/49.4–51.6%). At 30 weeks of age, cancer tissues and normal prostate glands were mixed in the experimental group (Fig. 2C); however, PC tissues with glandular structures were found in the control group (Fig. 2D). Furthermore, at 52 weeks of age, the area of cancer tissues was increased in the experimental group, although glandular PC tissues and normal glands still existed (Fig. 2E). In contrast, in the control group, undifferentiated cancer cells clearly appeared at 52 weeks of age (Fig. 2F). From our observations, we found that tumorigenesis only occurred in the ventral prostate lobe, not the dorsolateral lobe.

### Frequency of cancer cells

**Figure 3:**
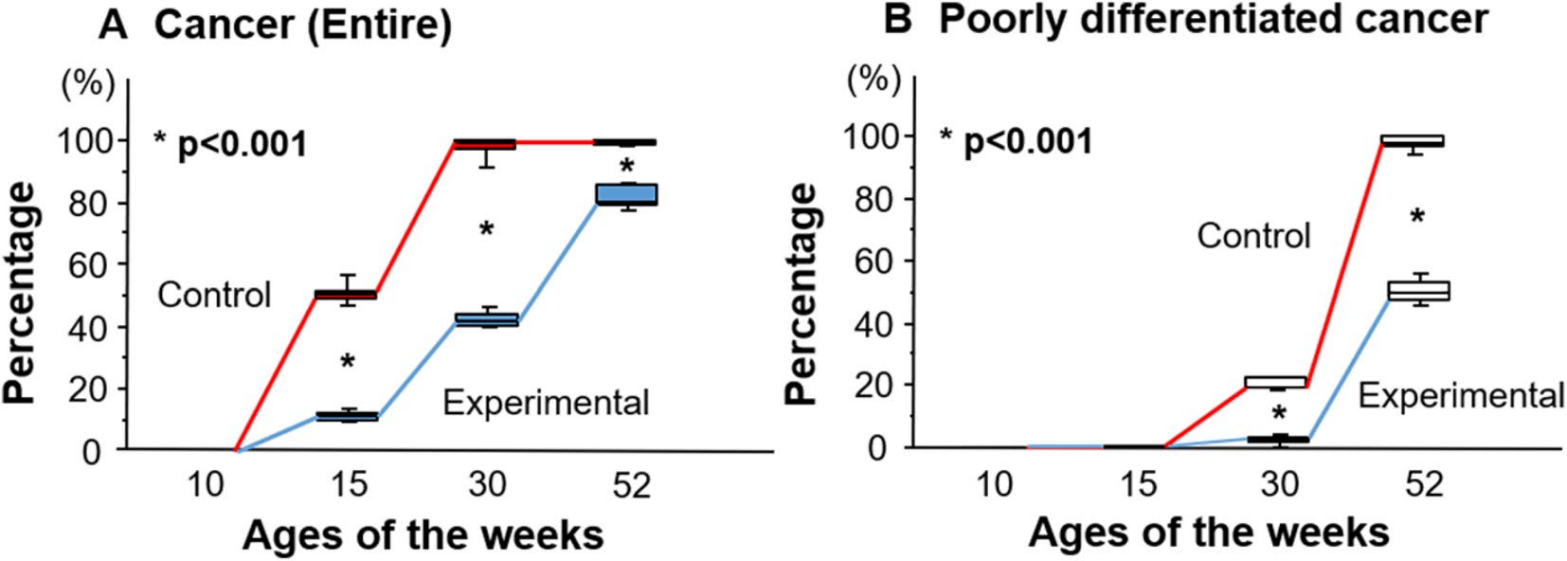
Percentage of cancer (**A**) and poorly differentiated cancer (**B**) at 10, 15, 30, and 52 weeks of age in control and experimental mice.

The changes in the percentage of cancer cells in the experimental and control groups are shown in Fig. 3A. The frequencies of cancer cells in experimental group were significantly lower compared to control group at 15, 30 and 52 weeks of age. On the other hand, as shown in Fig. 3B, there were no significant differences in poorly differentiated PC cells between the groups at 10 and 15 weeks of age. However, the percentage of poorly differentiated PC cells was significantly lower (p < 0.001) in the experimental group (2.7/1.8–3.4% and 49.9/47.5–52.7%) than in the control group (19.6/19.2–22.1% and 98.4/97.3–100.0%) at 30 and 52 weeks of age, respectively (Fig. 3B). Thus, at 52 weeks of age, although almost all cancer cells were judged as poorly differentiated in the control group, the frequency of poorly differentiated PC cells in the experimental group was nearly half that of cancer cells.

### Survival and Safety

In the control group, 2 of 15 mice (11.1%) died before 30 weeks of age, and 4 of 15 mice (26.7%) died from 31 to 52 weeks of age. In contrast, only one mouse (6.7%) died at 43 weeks of age in the experimental group. There was no injury, bite, or infection in any of the mice, including the dead mice. There was no significant difference in body weight or food intake between the control and experimental groups. There were no abnormal pathological findings in H&E-stained kidney and liver tissues in both of experimental and control group; however, we did not collect data on renal and liver function from blood or urine tests.

### Change of frequency of apoptotic cells

**Figure 4:**
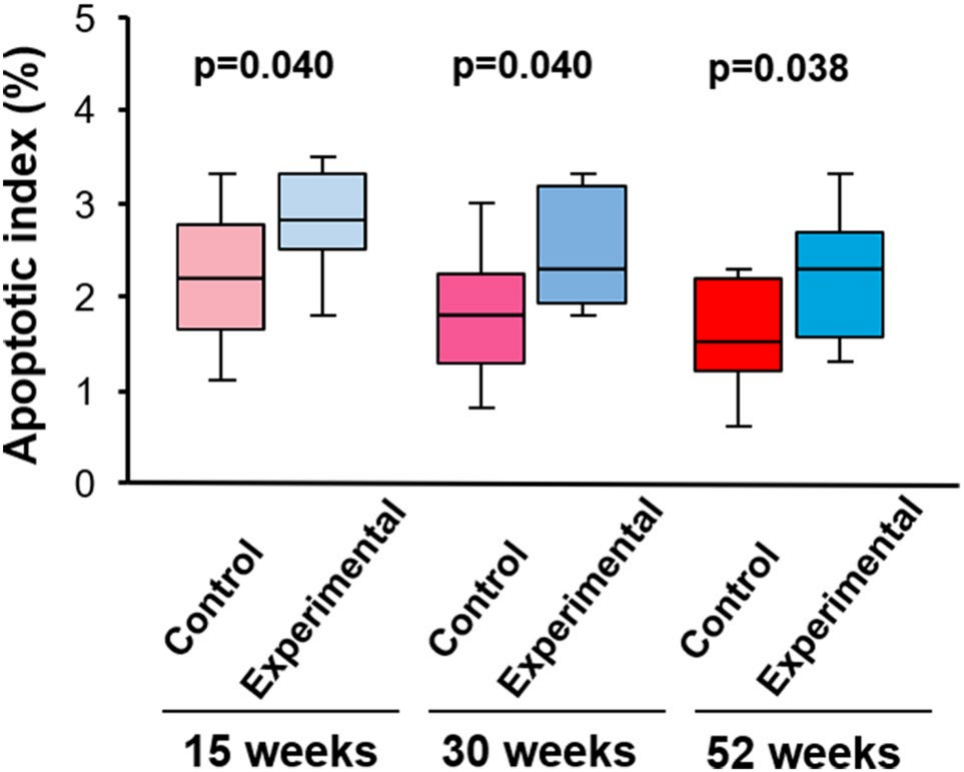
The percentage of apoptotic cells in control and experimental mice at 15, 30, and 52 weeks of age.

As shown in Fig. 4, at 15 weeks of age, AI in the experimental group (2.8/2.5–3.3%) was significantly higher (p = 0.040) than that in the control group (2.2/1.8–2.8). In addition, a similar significant difference was found at 30 and 52 weeks of age (p = 0.040 and 0.038, respectively; Fig. 4).

### Discussion

The present study demonstrated that the EP1 receptor antagonist delayed carcinogenesis and tumor growth in a PC animal model. Many investigators have suggested that COX-2 inhibitors are useful for the chemoprevention and treatment of malignancies in preclinical studies and clinical trials^30–32^. However, it should be noted that the addition of COX-2 inhibitor did not significantly affect the outcomes of randomized clinical trials of non-small cell lung cancer and colon cancer patients^33,34^. In PC, several in vivo and in vitro studies showed that anti-cancer effects including improvement of prognosis of COX-2 inhibitors were limited^35–38^. Thus, the chemopreventive and anti-cancer effects of COX-2 inhibitors in PC are still controversial. On the other hand, comprehensive regulation of PGE2 production by systematic administration of COX-2 inhibitors is speculated to lead to weakness of anti-cancer effects and increased risk of adverse events due to global prostanoid suppression^39^. In fact, COX-2 inhibitors are known to upregulate the risk of various visceral disorders, such as gastrointestinal and cardiovascular toxicities^40–42^. In addition, other investigators have suggested that inhibition of the EP receptor pathway is a more effective approach for improving the anti-cancer effects compared to treatment strategies using COX-2 inhibitors^43^. Based on these facts, we believe that more specific inhibition of PGE2 activity is necessary to improve the efficacy and safety of chemoprevention and treatment of PC patients. Finally, although there were no data on anti-cancer effects of COX-2 inhibitors in our PC mouse model, we conducted a preliminary investigation of the effects of the EP1 receptor inhibition.

Regarding the expression pattern and pathological roles of EP receptors in PC, in vitro studies showed that EP2 and EP4 receptors were expressed in PC-3 cells and in PC-3, DU145, LNCaP, and PrEC cells, respectively^44^. Other in vitro studies have also shown that EP2 and EP4 receptors are mainly expressed in PC cell lines, and overexpression of EP2 and EP4 receptors and reduced EP3 expression were observed in PC tissues^18,19^. Thus, these reports showed that the pathological significance of the EP1 receptor was minimal in PC. However, interestingly, inhibition of EP1 receptor signaling led to the suppression of proliferation in PC cell lines^45^. In addition, in an animal model, EP1 receptor-positive PC cells play a crucial role in cancer cell proliferation^20^. Moreover, in human PC tissues, EP1 receptor expression is significantly associated with Gleason score, TNM stage, and cancer cell proliferation^17^. Although there was no general agreement on the pathological roles of the EP1 receptor in PC, we selected the EP1 receptor antagonist according to the results obtained in PC cell lines, animal experiments, and human tissues.

The usefulness of treatment strategies by antagonists of each EP receptor has been reported in various types of malignancies; for example, the EP1 and EP2 receptors for breast cancer^46,47^, EP3 receptor for oral cancer^48^, and EP4 receptor for lung cancer and breast cancer^43,49,50^. On the other hand, regarding PC, the EP1 receptor antagonist (SC51322) showed anti-proliferative effects on cancer cells, whereas the EP2, EP3, and EP4 receptor antagonists did not^45^. Unfortunately, there is little information on the pro-apoptotic activity of EP1 receptor inhibitor in PC cells. However, oral intake of an EP1 antagonist was reported to have chemopreventive effects via stimulation of apoptosis without any side effects in a breast cancer animal model^46^. These previous findings support our results on chemopreventive effects, stimulative function of apoptosis, and safety of EP1 antagonist.

A limitation of this study is that the chemopreventive effects of other EP receptor antagonists have not been investigated. In addition to the EP1 receptor, in vitro and animal experiments have shown that the EP4 receptor is a potential therapeutic target for PC^51^. Furthermore, we previously reported that EP2 receptor- and EP3 receptor-expressing cancer stromal cells were positively associated with cancer cell progression and worse outcomes in patients with PC^16^. Thus, it is possible that EP2–EP4 antagonists may have chemopreventive and anti-cancer effects in in vivo studies. In recent years, a combination therapy of anti-PD-L1 antibody and EP4 antagonist enhanced anti-tumor growth effects and prolonged survival in mice inoculated with murine lymphoma cells^52^. Finally, we suggest further in vivo studies, including animal experiments, to discuss the usefulness, limitations, and safety of novel therapeutic strategies by inhibition of EP receptor pathways and of combined therapies with such treatments and conventional therapies in PC. In addition, detailed studies on the molecular and pharmacological mechanisms, serum and tissue levels, and downstream activities of the EP1 receptor antagonist in PC are critical to understand the mechanisms involved in its anti-cancer effects. Furthermore, studies designed to clarify the long-term effects of EP1 receptor expression on KIMAP are important to determine the safety and reliability of chemopreventive effects of the EP1R antagonist in PC.

Lastly, lymph node metastasis and visceral (lung and liver) metastases in the late-stage KIMAP have been reported (over 52 weeks)^22^. However, this study design did not exceed 52 weeks, and the frequencies of such metastases were not investigated in either the experimental or the control group; we acknowledge that this is a limitation of the current study.

## Conclusion

Our in vivo study using KIMAP demonstrated that an EP1 receptor antagonist delayed carcinogenesis and progression of PC. Induction of apoptosis was speculated to be associated with such chemopreventive effects. Finally, we concluded that inhibition of the EP1 receptor pathway by an EP1 antagonist is a novel chemopreventive strategy for PC.

## Supplementary Information

Supplementary Information.
