# Supplementary figures and images for: Oral administration of E-type prostanoid (EP) 1 receptor antagonist suppresses carcinogenesis and development of prostate cancer via upregulation of apoptosis in an animal model
Source: Sci Rep. 2021 Oct 13;11:20279. doi: 10.1038/s41598-021-99694-y (PMC8514456; doi:10.1038/s41598-021-99694-y)

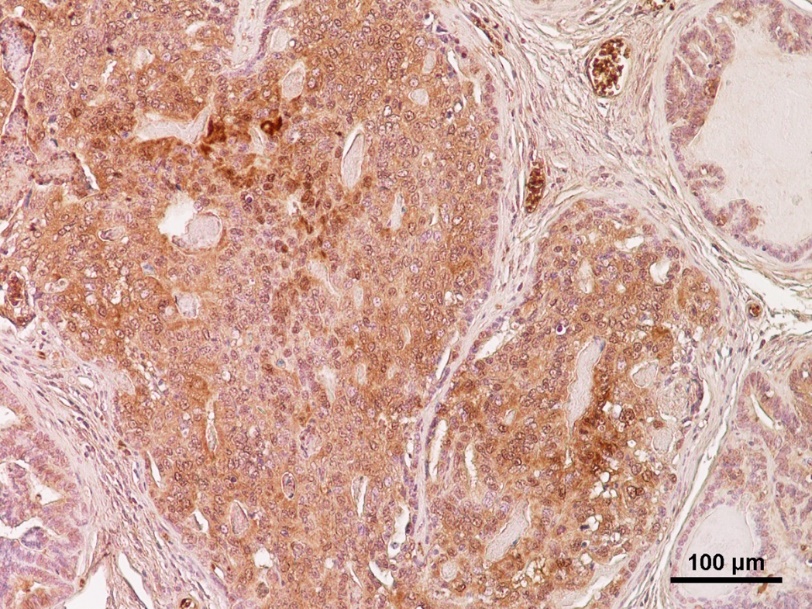


Figure S1. EP1 receptor expression in prostate cancer tissues of KIMAP.

Supplement: Supplementary file 1 — Supplementary Information. [file 41598_2021_99694_MOESM1_ESM.docx]
